# Phenolisation of the Sinus Tract in Recurrent Sacrococcygeal Pilonidal Sinus Disease: A Prospective Cohort Study

**DOI:** 10.7759/cureus.8129

**Published:** 2020-05-15

**Authors:** Akke Pronk, Michiel Vissink, Niels Smakman, Edgar Furnee

**Affiliations:** 1 Surgery, Diakonessenhuis, Utrecht, NLD; 2 Surgery, University Medical Center Groningen, Groningen, NLD

**Keywords:** phenolisation, pilonidal sinus, minimal invasive surgery, quality of life, recurrence, prospective cohort study

## Abstract

Purpose

Phenolisation is a minimally invasive treatment option in patients with primary pilonidal disease. However, most studies focus on patients with primary pilonidal sinus disease, while data of patients with recurrent pilonidal disease are very scarce. The purpose of this study was to evaluate phenolisation of the sinus tract in patients with recurrent pilonidal sinus disease after previous surgery for SPSD.

Methods

This single-center prospective cohort study included 60 patients with recurrent pilonidal disease. Loss of days of normal daily activities, surgical site infection, wound epithelization, quality of life, and complaints related to pilonidal disease were postoperatively assessed.

Results

A total of 57 patients (95%) were treated with phenolisation and the median loss of days of normal daily activities was 5.0 (1.0 - 12.0) days. Fifty-one patients (89.5%) resumed normal daily activities after two weeks. Surgical site infection occurred in five patients (8.8%). Compared to preoperative scores, quality of life was significantly higher 12 weeks postoperatively (p=0.014) and pain and itch scores were lower after six and 12 weeks (p ≤ 0.005). Wounds were completely healed in 45 of 51 patients (89.8%) who were available after 12 weeks of follow-up.

Conclusion

Phenolisation for recurrent pilonidal disease is safe with a median complete return to daily activities within five days and complete wound healing after three months in 90%. Therefore, phenolisation should be considered as a treatment option in patients with recurrent pilonidal sinus disease.

## Introduction

Sacrococcygeal pilonidal sinus disease (SPSD) has a high incidence [1]. Different treatment modalities are currently applied for SPSD. In the case of a tense, painful abscess, a simple incision and drainage would be sufficient in most cases. However, in patients with symptomatic chronic SPSD, excision with or without primary wound closure is very often applied as a definitive management procedure. As a result, wound dehiscence with longer wound healing time is a major problem after this type of surgery. Besides, recurrence is also an important issue in patients surgically treated for SPSD as recurrence rates up to 68% have been reported 10 years after surgery, depending on the type of surgery performed [2]. Repeated treatments in the case of recurrence with long recovery times could easily have a large impact on patients' quality of life. Therefore, recently published literature is more focused on minimally invasive treatment modalities such as phenol, laser or endoscopic treatments. In these minimally invasive treatments, wounds are smaller, and therefore, wound healing time is shorter.

Phenolisation is a minimally invasive treatment modality for patients with symptomatic primary chronic SPSD that has shown to be safe and effective in patients with primary SPSD. Two randomized trials comparing phenol application and excision for primary SPSD have been performed so far, showing lesser postoperative pain, faster return to normal daily activities, quicker wound healing as well as equal surgical site infection and recurrence rates after phenolisation [3-4]. Several additional cohort studies on phenol application on primary SPSD have been published showing comparable results. However, studies evaluating phenol application in patients with recurrent SPSD after previous surgical excision are very scarce with only two published cohort studies including 36 and 26 patients, respectively [5-6].

In this prospective cohort study, we evaluated phenolisation of the sinus tract in patients with recurrent SPSD after previously undergoing surgery for SPSD. 

## Materials and methods

Study design

This was a prospective single-center cohort study conducted in Diakonessenhuis, Utrecht, Netherlands. Patients were considered for participation in this study if they presented with SPSD at the department of surgery. Patients with recurrent SPSD after one or more previous surgical treatments for SPSD and age ≥ 18 years were eligible for inclusion in this study. Patients who presented with no or minimal symptoms related to SPSD, an abscess due to SPSD, a large network of subcutaneous sinus tracts, or primary SPSD (patients with these conditions are not eligible to undergo phenolisation) were excluded. This study was approved by the Medical Ethical Committee in Utrecht.

Surgical interventions and data collection

Data about body mass index, smoking, working position, quality of life (QoL), family history, duration of symptoms related to SPSD, and previous surgical intervention(s) for SPSD were prospectively collected for all patients before surgery. More detailed questions about symptoms related to SPSD (i.e. itching and pain) were evaluated; each symptom was scored on a six-point scale from 0 to 5 (ranging no complaints to daily complaints). A visual analogue scale (VAS), scored from 0 to 100 (worst to best), was used to measure the QoL.

Phenolisation of the recurrent pilonidal sinus was performed in the operation room. The anesthesiologist and patient decided, depending on their preferences, if the anesthesia was spinal or general. Antibiotics were not administered. Patients were positioned in a prone position and to optimize the view of the area of the natal cleft, plasters were used to separate the buttocks. After cleaning the natal cleft with antiseptic solution (Betadin 100 mg/mL), the area was covered with sterile dressings. The pit(s) of the sinus were probed to determine the direction of the sinus(es). All the pit(s) were excised as limited as possible. A curette was introduced in the excised pit(s) to clean the tract. All the hairs, debris, and granulation tissue were removed. Hemostasis was reached by using electrocautery and/or external compression. To protect the area of the natal cleft against phenol, a gauze was used to protect the anus and the skin was protected by Vaseline. After secure protection, liquid phenol (85%) was injected using syringes. The amount of injected phenol depended on the volume of the tract. After one minute, phenol was aspirated and a new same amount of phenol was injected once more, again for one minute. After a second time of aspiration, the remaining phenol was washed out with ethanol (70%) to neutralize remnants of phenol.

Data on the number of midline and off-midline pits, duration of operation, intraoperative complications, and presence of hair and the volume of the sinus were prospectively collected. Postoperative advice to all patients was to keep the buttocks clean and epilate the hair from the skin surrounding the wound. Patients were admitted the same day.

Patients were asked to complete a diary for two weeks postoperatively. Data with regard to complaints related to the treatment, i.e. fluid discharge, pain, irritation, itching, and burning sensation were scored using the six-point scale as mentioned previously. A VAS score was used to score pain from 0 to 100 (no pain to extremely painful). Patients also indicated every day if they used any painkillers and if they were able to perform normal daily activities, such as working or doing housekeeping work.

Subjective evaluation was obtained by a questionnaire two, six and 12 weeks after surgery containing items about patient’s satisfaction (disease scored as cured, improved, unchanged or worsened), complaints related to the phenol treatment (same complaints and scoring system as used in the diary), QoL (VAS) and regarding return to normal daily activities.

Two, six and 12 weeks after surgery, the wound was evaluated in the outpatient clinic by one of the investigators (EF or NS) through an assessment form, including wound closure (defined as complete epithelization of the skin), size of the wound(s) in three dimensions (in the case of no complete epithelization), and surgical site infection (SSI).

Statistical analysis

Data were analyzed using SPSS for Windows version 23.0 (SPSS Inc., Chicago, Illinois, USA). Values were reported as mean (± SD) or as median (interquartile range) in the case of continuous values, depending on whether the data were normally distributed or not. Categorical values were reported as frequencies and percentages of the total number of patients. Differences between continuous pre- and postoperative values were statistically analyzed by the paired-samples t-test. When data were not normally distributed, the Wilcoxon signed-rank test was performed. Differences were considered statistically significant with P-value < 0.050.

## Results

**Figure 1:**
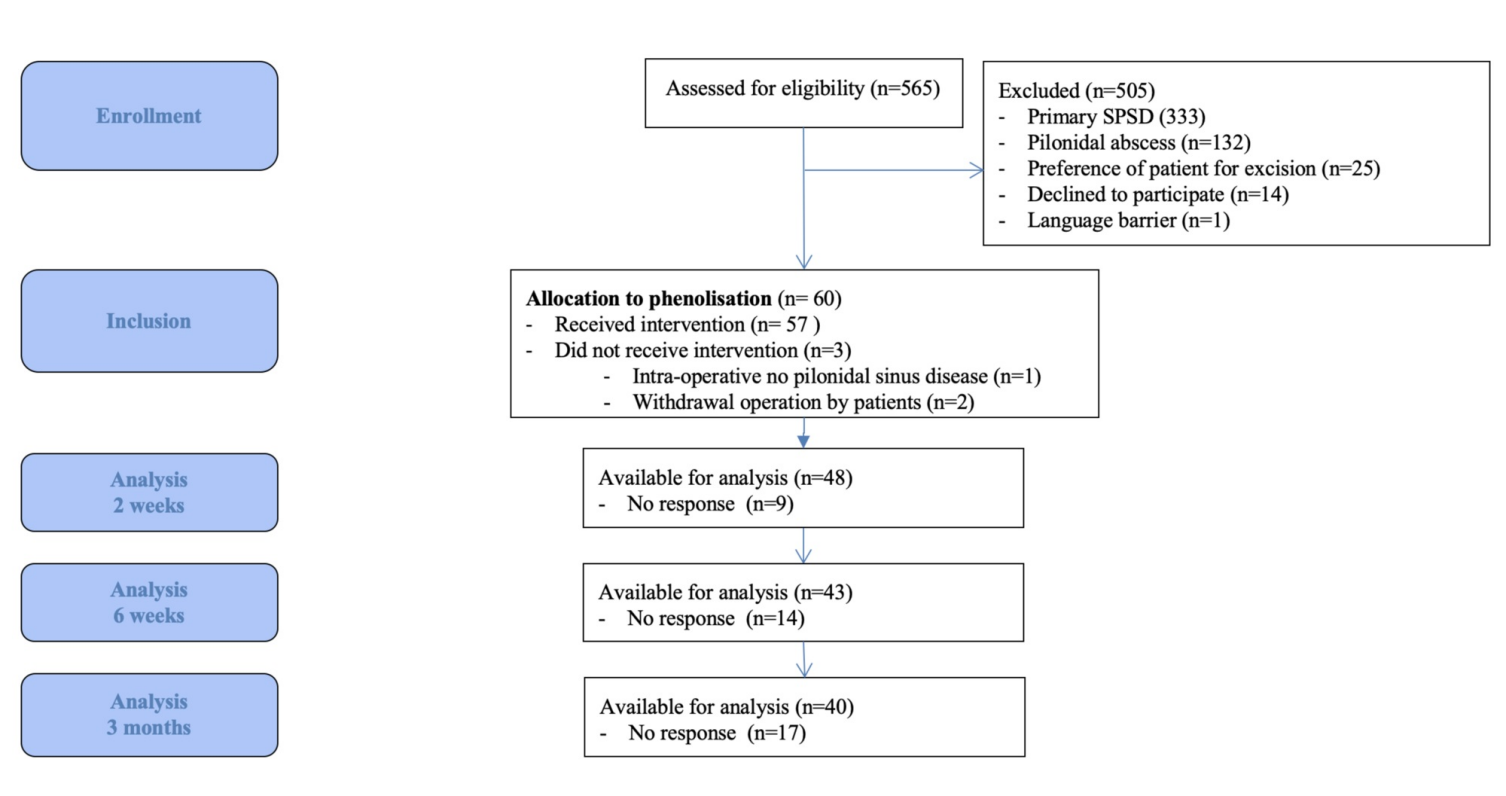
Flow Chart Consort Abbreviation: SPSD, sacrococcygeal pilonidal sinus disease. Abbreviation: SPSD, sacrococcygeal pilonidal sinus disease.

A total of 60 out of 565 patients who presented with SPSD at Diakonessenhuis between September 2013 and September 2017 were eligible for inclusion in the current study (Figure 1). Since two patients refused surgical treatment and one patient had no SPSD intra-operatively, 57 underwent phenolisation. Table 1 presents the baseline characteristics of the included patients. Previous surgery for SPSD was performed once in 52 patients (86.7%), twice in three patients (5.0%), three times in three patients (5.0%), four times in one patient (1.7%) and in one patient the number and kind of previous treatment(s) was unknown as this was performed in another hospital a long time ago. A total of 39 previous procedures (65.0%) were incision and drainage of an abscess, in 16 cases (26.7%) excision of the pilonidal sinus disease was performed and four previous procedures were phenolisation. In the remaining cases, the specific procedures were unknown. The median interval between the last surgical procedure and phenolisation for recurrence was 13 (5.8 to 41.3) months.

**Table 1:** Baseline Characteristics Values are reported as mean ± SD or median (interquartile range), unless otherwise stated.

	Phenolisation n = 60
Male sex (%)	50 (83.3)
Age (years)	29.2 (10.8)
Body mass index (kg/m^2^)	25.4 (4.0)
Smoking (%)	22 (36.7)
Family history of pilonidal sinus disease (%)	10 (16.7)
Working in sitting position (%)	37 (61.7)
Duration of preoperative symptoms (months)	17.4 (21.1)
Number of sinus pits midline	2.1 (1.4)
Patients with sinus pit(s) at right side of midline (%)	14 (23.3)
Patients with sinus pit(s) at left side of midline (%)	15 (25.0)

Peri-operative data

The mean duration of operation was 18.7 (6.4) minutes and there were no intra-operative complications. The mean volume of the sinus was 1.0 (1.0) mL. All patients could be discharged on the same day of surgery. Postoperatively, surgical side infections were seen in five patients (8.8%); one patient developed an abscess, and four patients cellulitis. None of the patients was readmitted. 

**Figure 2:**
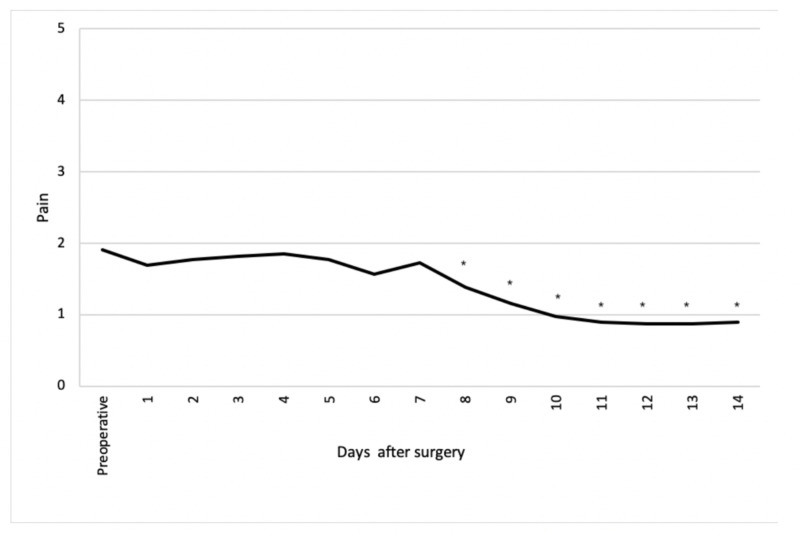
Pain at natal cleft Items scored on a six-point scale from 0 (no pain) to 5 (daily pain). *Items showing a statistically significant difference compared to preoperatively (P<0.05).

**Figure 3:**
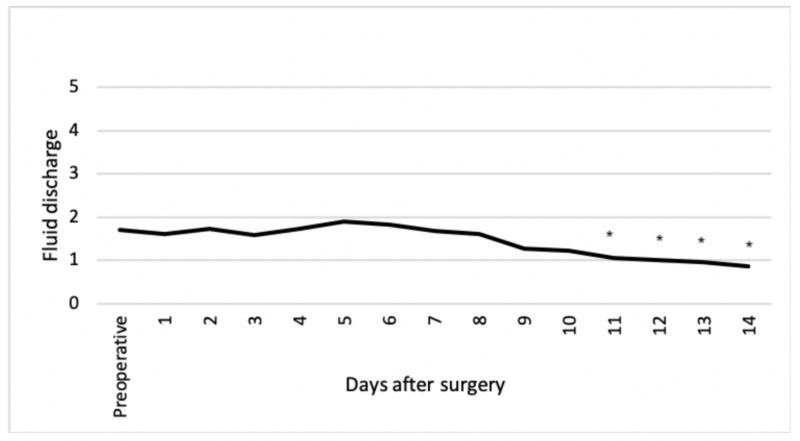
Fluid discharge at natal cleft Items scored on a six-point scale from 0 (no discharge) to 5 (daily discharge). *Items showing a statistically significant difference compared to preoperatively (P<0.05)

From eight days after surgery, pain was significantly less compared to preoperatively (Figure 2). In addition, fluid discharge was significantly less compared to preoperatively from day 11 after surgery (Figure 3). Itching, irritation and a burning sensation at the natal cleft was rarely present with a maximum score of one during the first two weeks after surgery.

Follow-up data

Figure 1 shows the number of patients available for follow-up two, six and 12 weeks after surgery. Median loss of days of normal daily activities was 5.0 (1.0 - 12.0) days. A total of 51 patients (89.5%) resumed normal daily activities after two weeks after surgery; after postoperative weeks 6 and 12, normal daily activities were resumed in 52 (91.2%) and 53 patients (93.0%) respectively.

The pain and itch scores were significantly lower two, six, and 12 weeks after surgery compared to preoperatively (Table 2). QoL dropped two weeks after surgery but was significantly higher 12 weeks after surgery compared to preoperatively (Table 2). A total of 36 patients (90.0%) reported SPSD as fully cured or significantly improved 12 weeks postoperatively compared to before the surgery. Two patients (5.0%) reported SPSD as unchanged and one patient (2.5%) as worsened. In addition to the 17 patients who did not respond (Figure 1), one patient (2.5%) did not complete this item in the questionnaire.

**Table 2:** Pre- and postoperative subjective and objective data Values are reported as median and interquartile range (IOR), unless otherwise stated. N/A; not applicable, N.R.; not reported, VAS; visual analogue scale, *Items scored on a six-point scale from 0 (no complaints) to 5 (daily complaints) **p-value compared with values two weeks after surgery

	Preoperative	2 weeks postoperative	p-value compared to preoperatively	6 weeks postoperative	p-value compared to preoperatively	12 weeks postoperative	p-value compared to preoperatively
Subjective data							
Pain (VAS, 0-100)	15.0 [6.0 – 36.5]	8.0 [0.50 – 19.0]	0.091	5.0 [1.0 – 10.0]	0.010	9.0 [0.0 – 19.0]	<0.001
Pain*	2.0 [1.0 – 3.0]	1.0 [0.0 – 2.0]	<0.001	0.0 [0.0 – 1.0]	<0.001	0.0 [0.0 – 2.0]	0.005
Itch*	2.0 [1.0 – 2.0]	1.0 [0.0 – 1.0]	<0.001	0.0 [0.0 – 1.0]	<0.001	0.0 [0.0 – 1.25]	<0.001
Fluid*	2.0 [1.0 – 2.5]	1.0 [0.0 – 1.0]	0.013	0.0 [0.0 – 1.0]	<0.001	0.0 [0.0 – 1.0]	0.248
Irritation*	N.R.	0.0 [0.0 – 1.0]	N/A	0.0 [0.0 – 1.0]	0.851**	0.0 [0.0 – 1.0]	0.074**
Burning sensation*	N.R.	0.0 [0.0 – 1.0]	N/A	0.0 [0.0 – 1.0]	0.546**	0.0 [0.0 – 1.0]	0.480**
Quality of life (VAS, 0-100)	73.0 [50.0 – 80.0]	66.0 [50.0 – 79.5]	0.182	75.0 [65.0 – 83.0]	0.072	75.0 [68.0 – 83.0]	0.014
Objective data							
Length of wound (mm)	N/A	8.0 [3.0 – 15.0]	N/A	3.1 [1.7 – 9.3]	0.019**	0.0 [0.0 – 8.4]	0.011**
Width of the wound (mm)	N/A	3.0 [2.0 – 5.0]	N/A	2.0 [1.0 – 4.8]	0.018**	0.0 [0.0 – 3.0]	0.030**
Depth of the wound (mm)	N/A	5.0 [2.0 – 10.3]	N/A	2.0 [1.0 – 9.0]	0.123**	0.0 [0.0 – 5.3]	0.059**

Complete wound healing was achieved in nine patients two weeks after surgery. Six weeks after surgery, the wound was completely healed in an additional of 26 patients and an additional 10 patients 12 weeks after surgery. In six patients, it was not possible to assess complete wound healing as those patients dropped out during follow-up before the wound was completely healed. So, complete anatomic wound healing was reached in 45 of 51 patients (89.8%) after 12 weeks of follow-up. The dimensions of the open wounds two, six, and 12 weeks after surgery are reported in Table 2.

## Discussion

This is, to our knowledge, the largest prospective cohort study reporting on the outcome of phenolisation for recurrent SPSD. The results showed a median of five days of loss of normal daily activities after phenolisation of the sinus tracts in patients with recurrent SPSD. Although quality of life dropped two weeks after surgery, it was significantly higher 12 weeks after surgery compared to the quality of life preoperatively. In addition, pain also significantly decreased 12 weeks after surgery. The operation wounds were completely healed in 90% of patients 12 weeks after surgery.

Comparing the loss of days of daily activities after phenolisation in patients with recurrent SPSD and patients with primary SPSD showed comparable results (five days vs. five days, respectively) [4]. Two previous studies investigated crystallized phenol in patients with recurrent SPSD. One study was retrospective, including 26 patients, and a cure rate of 92.3% was achieved [5]. Another study had a prospective study design and included 36 patients [6]. Patients had minimal pain and most patients reported no complaints. Wounds were completely healed after a median of 48 days. The loss of daily activities was not reported in both studies.

Comparing the loss of days of daily activities after phenolisation with excision in patients with recurrent SPSD showed different results. Two randomized controlled trials compared Limberg flap and Karydakis flap in patients with recurrent SPSD. One study included 71 patients; treating 37 patients with Limberg flap and 34 patients with Karydakis flap. Median loss of days of daily activities was eight (range 6-12) and 17 (range 14-20) days, respectively [7]. The second trial included 120 patients; 60 patients were treated with Karydakis flap, and 60 with Limberg flap. Time to return to work was 20 (± 6.01) and 22.35 (± 4.8) days, respectively [8]. So, excision of the pilonidal sinus results in longer times to return to normal daily activities compared to the phenolisation technique as was shown in the current study. There are also other minimally invasive techniques to treat SPSD available, including the endoscopic pilonidal sinus treatment (EPSiT) and laser technique. EPSiT has been investigated in a prospective multicenter study including 122 patients with recurrent SPSD [9]. Return to work was after three days, even faster compared to the phenolisation technique. The laser technique was also evaluated in a mixed group with both primary and recurrent SPSD by Pappas et al. [10] In this study, 92.8% of all patients resumed daily activities immediately, a little faster compared to the results in the current study for the phenolisation technique.

The benefits of phenolisation are due to the minimal invasive character; smaller wounds leading to less postoperative pain and quicker skin epithelization at the natal cleft. The benefits of phenolisation have been reported before in patients treated for primary SPSD, but not for patients who presented with recurrent SPSD [4]. Our current study showed that phenolisation of the sinus tract in patients with recurrence SPSD is safe as no major perioperative complications occurred along with only an SSI rate of 9%. Two other studies reported results of phenolisation in patients with recurrent SPSD reported an SSI rate of 15.4% and 0% without any major complications [5-6]. The results of the current study also proved the clear benefits of smaller wounds with the phenolisation technique in recurrent SPSD; patients experienced less pain with a quick return to normal daily activities and complete wound healing achieved in almost 90% of patients 12 weeks after surgery.

SPSD has a high prevalence and recurrence is still a major problem in patients with SPSD. Currently, no consensus exists on the best treatment modality for recurrent SPSD. Almost no data have been reported on the outcome of the different traditional treatment modalities in patients with recurrent SPSD. In addition, there is a trend towards minimally invasive techniques in patients with primary SPSD, including phenolisation, laser, and endoscopic treatments [9-10]. However, in almost none of the reported studies, is there focus on treatment with a minimally invasive modality in patients with recurrent SPSD. Therefore, we performed the current study on a minimally invasive technique in recurrent SPSD, i.e. phenolisation of the sinus tract. The results of the current study on phenolisation in recurrent SPSD are promising and therefore, we have increasingly utilized the minimally invasive treatment with phenolisation, also in patients with recurrent SPSD.

Although this is, as far as we know, the largest prospective study reporting on the outcome of phenolisation in recurrent SPSD, there are some limitations that need to be addressed. Loss of patients during follow-up was relatively high in the current study. The relative high drop-out rate could be explained by the fact that the patients in the current study mainly include the young and healthy working population. Patients without symptoms easily cancel their appointments because of their focus on work and social life, especially if the reasons for complaints are not present anymore. This has probably resulted in worse results in the current study as it is more likely that patients with persisting symptoms respond to follow-up. In addition, the recurrence rate was not reported in the current research. Since the aim of the current study was to focus on the short-term benefits of the minimally invasive phenolisation technique, especially on loss of days of normal daily activity, the duration of follow-up was 12 weeks. Therefore, we were not able to report the reliable recurrence rate as this would require a follow-up of at least 12 months.

## Conclusions

In conclusion, this is the largest prospective cohort study on phenolisation of the sinus tract in patients with recurrent pilonidal sinus disease showing less pain, improvement of quality of life with only five days of loss of normal daily activities and complete wound healing in 90% of patients after 12 weeks. In addition, phenolisation in recurrent SPSD appeared to be safe without the occurrence of major complications. Since the results of this study are very promising, surgeons should also consider phenolisation of the sinus tracts as a treatment option in patients with recurrent SPSD. 
